# Antimicrobial Peptide Exposure Selects for Resistant and Fit Stenotrophomonas maltophilia Mutants That Show Cross-Resistance to Antibiotics

**DOI:** 10.1128/mSphere.00717-20

**Published:** 2020-09-30

**Authors:** Paula Blanco, Karin Hjort, José L. Martínez, Dan I. Andersson

**Affiliations:** a Centro Nacional de Biotecnología, CSIC, Madrid, Spain; b Department of Medical Biochemistry and Microbiology, Uppsala University, Uppsala, Sweden; CDC

**Keywords:** *Stenotrophomonas*, antibiotic resistance, antimicrobial peptides, cross-resistance, drug resistance evolution

## Abstract

Antimicrobial peptides (AMPs) are essential components of the innate immune system and have been proposed as promising therapeutic agents against drug-resistant microbes. AMPs possess a rapid bactericidal mode of action and can interact with different targets, but bacteria can also avoid their effect through a variety of resistance mechanisms. Apart from hampering treatment by the AMP itself, or that by other antibiotics in the case of cross-resistance, AMP resistance might also confer cross-resistance to innate human peptides and impair the anti-infective capability of the human host. A better understanding of how resistance to AMPs is acquired and the genetic mechanisms involved is needed before using these compounds as therapeutic agents. Using experimental evolution and whole-genome sequencing, we determined the genetic causes and the effect of acquired *de novo* resistance to three different AMPs in the opportunistic pathogen Stenotrophomonas maltophilia, a bacterium that is intrinsically resistant to a wide range of antibiotics. Our results show that AMP exposure selects for high-level resistance, generally without any reduction in bacterial fitness, conferred by mutations in different genes encoding enzymes, transporters, transcriptional regulators, and other functions. Cross-resistance to AMPs and to other antibiotic classes not used for selection, as well as collateral sensitivity, was observed for many of the evolved populations. The relative ease by which high-level AMP resistance is acquired, combined with the occurrence of cross-resistance to conventional antibiotics and the maintained bacterial fitness of the analyzed mutants, highlights the need for careful studies of S. maltophilia resistance evolution to clinically valuable AMPs.

**IMPORTANCE** Stenotrophomonas maltophilia is an increasingly relevant multidrug-resistant (MDR) bacterium found, for example, in people with cystic fibrosis and associated with other respiratory infections and underlying pathologies. The infections caused by this nosocomial pathogen are difficult to treat due to the intrinsic resistance of this bacterium against a broad number of antibiotics. Therefore, new treatment options are needed, and considering the growing interest in using AMPs as alternative therapeutic compounds and the restricted number of antibiotics active against S. maltophilia, we addressed the potential for development of AMP resistance, the genetic mechanisms involved, and the physiological effects that acquisition of AMP resistance has on this opportunistic pathogen.

## Abstract (precis)

Stenotrophomonas maltophilia is an increasingly relevant multidrug-resistant (MDR) bacterium found, for example, in people with cystic fibrosis and associated with other respiratory infections and underlying pathologies. The infections caused by this nosocomial pathogen are difficult to treat due to the intrinsic resistance of this bacterium against a broad number of antibiotics. Therefore, new treatment options are needed, and considering the growing interest in using AMPs as alternative therapeutic compounds and the restricted number of antibiotics active against S. maltophilia, we addressed the potential for development of AMP resistance, the genetic mechanisms involved, and the physiological effects that acquisition of AMP resistance has on this opportunistic pathogen.

## INTRODUCTION

The development of bacterial resistance to conventional antibiotics has prompted the search for new antimicrobial compounds, and antimicrobial peptides (AMPs) are potential candidates for therapeutic use due to their potent and broad-spectrum bactericidal activity (1). AMPs are diverse, short amphipathic, typically positively charged peptides that are produced by organisms in all kingdoms of life. In higher organisms, AMPs play an important role in the innate immune system and protect the host against microbial pathogens and infections (2) by directly killing bacteria and by acting as immunomodulators (3).

The antibacterial action of most AMPs relies mainly on their interaction between the positively charged peptide and the negatively charged membrane molecules, leading to pore formation, membrane permeabilization, and cell lysis (4, 5). Membrane permeabilization can also result in the translocation of certain AMPs into the cytoplasm, where they exert their action by interfering with key cellular processes, such as DNA and protein synthesis (5). This bactericidal activity makes AMPs promising candidates for use in the treatment of bacterial infections, and several of them are currently under clinical development or undergoing clinical trials (6), but to date only a few AMPs have been approved for clinical use. Among them, LL-37, the only cathelicidin with human origin, is used for the healing of leg ulcers (7) and is presently being evaluated in a phase II clinical trial for the treatment of diabetic foot ulcers (ClinicalTrials registration number NCT04098562). Similarly, polymyxins, a well-characterized group of AMPs introduced in the 1950s, have been recovered as last-resort drugs for the treatment of drug-resistant Gram-negative pathogens (8, 9).

Despite the initial thought that evolution of resistance to AMPs was improbable because of their rapid bactericidal effects and their multiple targets, studies have reported that bacteria are able to escape their effect through several types of resistance mechanisms, including modification of the bacterial outer membrane, exogenous peptide neutralization, degradation by proteases, and active efflux, among others (10). The acquisition of AMP resistance is of concern since bacteria could also develop cross-resistance against the host-defense peptides of the human immune system, together with cross-resistance to antibiotics (11). In this context, study of the acquisition of AMP resistance and the mechanisms involved, even before AMPs are used in clinics, is crucial in order to evaluate the risk of resistance emergence.

S. maltophilia is an important Gram-negative opportunistic pathogen associated with several clinical syndromes, such as respiratory infections in immunocompromised patients and in subjects that present a previous pathology, including cystic fibrosis (CF) or cancer (12). This bacterium exhibits low susceptibility to a wide range of antibiotics, including co-trimoxazole, quinolones, and cephalosporins. Because of the low susceptibility of S. maltophilia to antibiotics, which mainly relies on genes coding for antibiotic-inactivating enzymes and MDR efflux pumps located on the chromosome, the therapeutic options for the treatment of this bacterium are limited (13). Co-trimoxazole is the drug of choice for treating S. maltophilia infections, followed by quinolones. More recently, tigecycline, alone or in combination, has also been proposed as an alternative when the former antibiotics are not useful (14). Notably, mutants that overexpress the SmeDEF efflux pump can be selected and are cross-resistant to these three antibiotics (15–17), a cumbersome situation that requires the identification of novel antimicrobials for treating S. maltophilia infections. A few investigations have shown that AMPs are active against S. maltophilia (18–21), but no studies have explored the likelihood for emergence of resistance, the genetics behind resistance evolution, or the potential cross-resistance to antibiotics.

Here, we characterized the capability of S. maltophilia to acquire resistance to three structurally different AMPs from diverse origins, namely the two cathelicidins LL-37 and PR-39, which are produced by two of the hosts that S. maltophilia can infect (humans and pigs, respectively), and the polymyxin colistin. To this end, we combined serial-passage experiments in the presence of progressively increasing AMP concentrations in the mammalian ionic environment medium (MIEM), followed by whole-genome sequencing (WGS) to identify the genetic changes involved. The effects caused by prolonged exposure to AMPs on bacterial fitness and susceptibility to conventional antibiotics were assessed.

## RESULTS

### S. maltophilia experimental evolution in the presence of AMPs.

To elucidate if S. maltophilia can acquire resistance to AMPs by mutation, experimental evolution was carried out by daily serial passages in the presence of stepwise increasing concentrations of LL-37, PR-39, or colistin for 25 days (see Fig. S1 in the supplemental material). After this period, the MICs of each peptide in every lineage (8 independent lineages for each AMP) were determined (Table 1). In the case of LL-37, the concentrations during the experiment increased 2- to 3-fold in the evolved lineages. This modest increment suggests that the capability of S. maltophilia for adaptation to high concentrations of this human-derived peptide is lower than that for the other tested AMPs (see below). Unlike with LL-37, S. maltophilia reached high-level resistance to the porcine peptide PR-39, whose concentrations at the end of the evolution experiment were 17- to 25-fold higher for the evolved lineages. At the end of the experiment, the MICs of PR-39 increased at least 8-fold in comparison with that for the wild-type (wt) strain. Finally, during evolution in the presence of colistin, the concentration was increased 4- to 6-fold in the different lineages. As in the case of PR-39, the MICs of colistin at the end of the experiment increased 8-fold or more. The susceptibilities to the other two peptides not used for the evolution experiment were also assessed for all the evolved populations. As shown in Table 1, all of the LL-37 populations displayed cross-resistance to colistin, and two of them to PR-39. The populations evolved in the presence of the porcine cathelicidin showed a low-susceptibility phenotype to both LL-37 and colistin. Finally, half of the colistin-evolved populations displayed cross-resistance toward LL-37 or PR-39. To test the stability of the resistance phenotype, the 24 resistant mutants were serially passaged for approximately 50 generations in the absence of AMPs on lysogeny broth (LB) plates. Out of the 24 mutants, 22 showed a stable phenotype, whereas two (one LL-37- and one PR-39-selected clone) showed a reduction in resistance. Importantly, the control populations that evolved in the absence of any compound maintained the same susceptibility as that of the parental strain D457 to all tested peptides, showing that the serial passage procedure by itself does not select for resistance. These results show that S. maltophilia can acquire high-level resistance to LL-37, PR-39, and colistin and that the resistance phenotype is associated with cross-resistance to other AMPs.

**TABLE 1:** Susceptibility of resistant populations of S. maltophilia to AMPs

Strain	Passaged with	Initial concn (mg/liter)	Final concn achieved during serial passage (mg/liter)	MIC (mg/liter) of:			Isolated clone
				LL-37	PR-39	Colistin	
D457				100	7.5	2.5	
DA61805	MIEM			100	7.5	2.5	DA61861
DA61806	MIEM			100	7.5	2.5	DA61862
DA61807	MIEM			100	7.5	2.5	DA61863
DA61808	MIEM			100	7.5	2.5	DA61864
DA61715	LL-37	50	112.5	>200	60	10	DA61754
DA61716	LL-37	50	112.5	>200	7.5	5	DA61758
DA61717	LL-37	50	112.5	>200	7.5	>20	DA61759
DA61718	LL-37	50	112.5	200	7.5	>20	DA61764
DA61719	LL-37	50	168.75	>200	7.5	>20	DA61765
DA61720	LL-37	50	112.5	>200	7.5	>20	DA61770
DA61721	LL-37	50	112.5	200	>60	>20	DA61771
DA61722	LL-37	50	168.75	200	7.5	>20	DA61776
DA61723	PR-39	2	34.2	200	>60	20	DA62005
DA61724	PR-39	2	34.2	200	>60	20	DA61990
DA61725	PR-39	2	34.2	200	>60	20	DA62006
DA61726	PR-39	2	51.3	200	>60	20	DA61991
DA61727	PR-39	2	34.2	200	>60	20	DA61992
DA61728	PR-39	2	34.2	200	>60	20	DA61993
DA61729	PR-39	2	51.3	200	>60	>20	DA61994
DA61730	PR-39	2	51.3	200	>60	20	DA61995
DA61789	Colistin	0.25	1.59	100	7.5	>20	DA61947
DA61790	Colistin	0.25	1.59	100	7.5	20	DA61859
DA61791	Colistin	0.25	1.06	200	30	>20	DA61948
DA61792	Colistin	0.25	1.59	100	7.5	>20	DA61860
DA61793	Colistin	0.25	1.06	100	30	>20	DA62004
DA61794	Colistin	0.25	1.59	200	7.5	>20	DA61989
DA61795	Colistin	0.25	1.59	200	30	>20	DA61949
DA61796	Colistin	0.25	1.59	>200	30	>20	DA61950

10.1128/mSphere.00717-20.1 FIG S1 Evolution of AMP resistance in Stenotrophomonas maltophilia over 25 days. The graphs represent the daily concentrations in mg/litter of LL-37 (A), PR-39 (B), or colistin (C) to which the different S. maltophilia populations were exposed to during the 25-day evolution period. Download FIG S1, TIF file, 0.8 MB. Copyright © 2020 Blanco et al. 2020 Blanco et al. This content is distributed under the terms of the Creative Commons Attribution 4.0 International license.

### Genetic changes identified after AMP evolution.

One single colony from each of the eight peptide-evolved populations, as well as four colonies from the control experiments, were isolated for further studies. First, the susceptibility toward the AMP in which the evolution experiment was performed was tested for the selected clones. As shown in Table 2, all of the isolated clones displayed similar MICs to those of the evolved populations from which they derived. The isolated colonies from the control experiment did not show increased MICs of LL-37, PR-39, or colistin. The genomes of each of these clones were sequenced by WGS with the aim of identifying the mutations responsible for the reduced susceptibility to AMPs. All of the genetic changes found in the evolved clones are shown in Table 3. Mutations were found in genes and in intergenic regions, and the latter suggested that the alteration of the expression of some genetic determinants might contribute to the observed resistance phenotype.

**TABLE 2:** Susceptibility of resistant clones of S. maltophilia to AMPs

LL-37 clone^*a*^	MIC of LL-37 (mg/liter)	PR-39 clone	MIC of PR-39 (mg/liter)	Colistin clone	MIC of colistin (mg/liter)
D457 (wt)	100		7.5		2.5
DA61754	>200	DA62005	>60	DA61947	>20
DA61758	>200	DA61990	>60	DA61859	>20
DA61759	200	DA62006	>60	DA61948	>20
DA61764	>200	DA61991	>60	DA61860	>20
DA61765	>200	DA61992	>60	DA62004	20
DA61770	>200	DA61993	>60	DA61989	>20
DA61771	200	DA61994	>60	DA61949	>20
DA61776	>200	DA61995	>60	DA61950	>20

a wt, wild type.

**TABLE 3:** Mutations identified by WGS in the clones isolated after serial passage in absence and presence of AMPs

Strain	Serially passaged with	Gene(s) and product(s)^*a*^	Location	Type^*b*^	Change^*c*^	Potential contribution to resistance^*d*^	Reference
DA61861	No AMP	IGR *smd_0007* (hypothetical protein) and *smd_0008* (periplasmic binding protein TonB)	10561	SNP			
		*smd_2232* (glucan 1,4-alpha-glucosidase)	2484187	SNP	Arg468Pro		
DA61862	No AMP	IGR *smd_3461* (Xaa-Pro dipeptidase) and *smd_3462* (Xaa-Pro aminopeptidase)	3869293	Del 6 nt			
DA61863	No AMP	IGR *smd_2831* (LacI family transcriptional regulator) and *smd_2832* (BolA family transcriptional regulator)	3154404	SNP			
DA61754	LL-37	IGR *btuE2* (glutathione peroxidase) and *smd_2762* (hypothetical protein)	3077698	SNP		Unknown	
		*smd_3056* (SIMPL domain-containing protein)	3395040	SNP	Synonymous	Unknown	
DA61758	LL-37	*mraW* (rRNA small subunit methyltransferase H)	733802	Del 23 nt	Arg170fs	Modification of the expression of resistance-related genes through 16S rRNA methylation	42, 43
		*smd_0008* (periplasmic binding protein TonB)	10820	Del 27 nt	Pro68_Pro76 Del	Reduced interaction or uptake of LL-37	47 – 49
DA61759	LL-37	*mraW* (rRNA small subunit methyltransferase H)	733796	Del 23 nt	Gln171fs	Modification of the expression of resistance-related genes through 16S rRNA methylation	42, 43
DA61764	LL-37	*smd_0947* (putative autotransporter protein)	1068327	Del 4 nt	Ala3363fs	Unknown	
		*rluD* (ribosomal large subunit pseudouridine synthase D)	3732065	SNP	Val130Glu	Unknown	
DA61765	LL-37	*mraW* (rRNA small subunit methyltransferase H)	734100	Del 11 nt	Asn274fs	Modification of the expression of resistance-related genes through 16S rRNA methylation	42, 43
DA61770	LL-37	*hydG* (type IV fimbriae expression regulatory protein PilR)	3741629	SNP	Asp223Glu	Regulation of the type IV pilus protein PilA and alteration of membrane permeability	33, 34
DA61771	LL-37	*smd_0512* (asparagine synthetase)	582653	SNP	Asp489Gly	Unknown	
DA61776	LL-37	*smd_1285* (mercury ion transmembrane transporter)	1434096	Del 1 nt	Val49fs	Unknown	
DA62005	PR-39	*sspB* (stringent starvation protein B)	1607077	SNP	Leu19Pro	Regulation of proteases that might inactivate PR-39	50, 52, 53
		IGR *smd_1828* (cytochrome *c*) and *smmG* (Co/Zn/Cd efflux system MFP)	2018638	SNP		Potential extrusion of PR-39	15, 16, 57
DA61990	PR-39	*sspB* (stringent starvation protein B)	1607179	SNP	Val53Gly	Regulation of proteases that might inactivate PR-39	50, 52, 53
		IGR *smd_1828* (cytochrome *c*) and *smmG* (Co/Zn/Cd efflux system MFP)	2018638	SNP		Potential extrusion of PR-39	15, 16, 57
DA62006	PR-39	*sspB* (stringent starvation protein B)	1607471	Ins 1 nt	Stop151Ile	Regulation of proteases that might inactivate PR-39	50, 52, 53
		IGR *smd_1828* (cytochrome *c*) and *smmG* (Co/Zn/Cd efflux system MFP)	2018638	SNP		Potential extrusion of PR-39	(15, 16, 57)
DA61991	PR-39	*sspB* (stringent starvation protein B)	1607179	SNP	Val53Ala	Regulation of proteases that might inactivate PR-39	50, 52, 53
		IGR *smd_1828* (cytochrome *c*) and *smmG* (Co/Zn/Cd efflux system MFP)	2018638	SNP		Potential extrusion of PR-39	15, 16, 57
DA61992	PR-39	*sspB* (stringent starvation protein B)	1607179	SNP	Val53Gly	Regulation of proteases that might inactivate PR-39	50, 52, 53
DA61993	PR-39	*sspB* (stringent starvation protein B)	1607322	Del 5 nt	Gln102fs	Regulation of proteases that might inactivate PR-39	50, 52, 53
		IGR *smd_1828* (cytochrome *c*) and *smmG* (Co/Zn/Cd efflux system MFP)	2018638	SNP		Potential extrusion of PR-39	15, 16, 57
DA61994	PR-39	IGR *smd_1828* (cytochrome *c*) and *smmG* (Co/Zn/Cd efflux system MFP)	2018638	SNP		Potential extrusion of PR-39	15, 16, 57
		*ppa* (inorganic pyrophosphatase)	3912958	SNP	Gly2Asp	Restoration of the inorganic phosphate ions flow	10, 79
DA61995	PR-39	*sdhA* (succinate dehydrogenase flavoprotein subunit)	1909333	SNP	Asp239Gly	Proper functioning of the ETC	59 – 62
		IGR *smd_1828* (cytochrome *c*) and *smmG* (Co/Zn/Cd efflux system MFP)	2018638	SNP		Potential extrusion of PR-39	15, 16, 57
DA61947	Colistin	*smd_0260* (hypothetical protein)	314063	SNP	Arg76Cys	Unknown	
		*lptB* (lipopolysaccharide ABC transporter)	1154448	SNP	Thr179Pro	Alteration of the membrane LPS content	29
DA61859	Colistin	*wbiI* (polysaccharide biosynthesis protein)	2036869	SNP	Leu41Pro	Modification of the cell wall/LPS configuration	31, 32
		*smd_2876* (hypothetical protein)	3197529	SNP	Pro163Ser	Unknown	
DA61948	Colistin	*lptB* (lipopolysaccharide ABC transporter)	1154448	SNP	Thr179Pro	Alteration of the membrane LPS content	29
		*smd_2395* (outer membrane receptor for ferric coprogen and ferric-rhodotorulic acid)	2656970	SNP	Asp3Ala	Unknown	
		IGR between *btuE2* (glutathione peroxidase) and *smd_2762* (hypothetical protein)	3077698	SNP		Unknown	
DA61860	Colistin	*ftsW* (cell division protein FtsW)	740818	SNP	Leu163Val	Modification of the peptidoglycan content	30, 65
		*lptB* (lipopolysaccharide ABC transporter)	1154263	SNP	Stop240Trp	Alteration of the membrane LPS content	29
		IGR *suhB* (inositol-1-monophosphatase) and *htpX* (probable protease HtpX homolog)	3216769	SNP		Unknown	
		IGR *suhB* (inositol-1-monophosphatase) and *htpX* (probable protease HtpX homolog)	3216771	SNP		Unknown	
DA62004	Colistin	*sodB* (superoxide dismutase)	1612533	SNP	Stop193Cys	Defense against hydroxyl radical production by colistin	80 – 82
		*rpfG* (response regulator)	2232601	Ins 6 nt	Glu181_Thr182 Ins ArgGlu	Alteration of expression of membrane/LPS-related genes or alleviation of cellular damage	39, 40
		IGR *btuE2* (glutathione peroxidase) and *smd_2762* (hypothetical protein)	3077698	SNP		Unknown	
DA61989	Colistin	*sodB* (superoxide dismutase)	1612533	SNP	Stop193Cys	Defense against hydroxyl radical production by colistin	80 – 82
DA61949	Colistin	*phoQ* (sensor protein PhoQ)	315083	SNP	Ala30Val	Alteration of LPS modification-related genes	35 – 38
		*crp* (cyclic AMP receptor protein)	4370898	SNP	Arg149Gly	Alteration of expression of membrane/LPS-related genes or alleviation of cellular damage	41, 83
DA61950	Colistin	*smd_0260* (hypothetical protein)	314136	SNP	Asp100Gly	Unknown	

a IGR, intergenic region; MFP, membrane fusion protein.

b SNP, single-nucleotide polymorphism; Del, deletion; Ins, insertion; fs, frameshift.

c Stop, stop codon.

d ETC, electron transport chain; LPS, lipopolysaccharide.

The bacteria that evolved in the absence of any AMP served as a control to identify any potential mutations that might be involved in medium adaptation during the serial passage in MIEM medium and which therefore were not associated with the resistance phenotype. In these clones, four different mutations were found, including single-nucleotide polymorphisms (SNPs) in a glucan 1,4-alpha-glucosidase-coding gene (*smd_2232*) and in the intergenic sequence between the genes coding for a hypothetical protein (*smd_0007*) and a periplasmic binding protein TonB (*smd_0008*) in DA61861. A deletion in the intergenic region between an Xaa-Pro dipeptidase-coding gene (*smd_3461*) and an Xaa-Pro aminopeptidase-coding gene (*smd_3462*) was present in DA61862. Finally, DA61863 had an SNP between the two genes *smd_2831*, encoding a LacI family transcriptional regulator, and *smd_2832*, which encodes a BolA family transcriptional regulator. None of the predicted −10 or −35 boxes of the mentioned genes were affected by these mutations (Table 3).

The eight LL-37-resistant clones had 11 mutations in total, with at least one mutation per clone, except for three clones that acquired two mutations each, including one in the intergenic region between a glutathione peroxidase-coding gene (*btuE2*) and a hypothetical protein-coding gene (*smd_2762*) in DA61654. This mutation was found in the predicted −10 box of the *smd_2762* promoter region, suggesting that the expression of this gene of unknown function might be altered. The only common change was found in *mraW*, encoding a rRNA small-subunit methyltransferase H, which was mutated in three of the clones (DA61458, DA61459, and DA61465). The mutated gene had deletions of 23 and 11 nucleotides, leading to a frameshift, and consequently to a truncated protein. The remaining LL-37-resistant clones acquired mutations in other elements, such as genes encoding a periplasmic binding protein, TonB (*smd_0008*), in DA61758; a putative autotransporter protein (*smd_0947*) and a ribosomal large-subunit pseudouridine synthase D (*rluD*) in DA61764; a type IV fimbriae expression regulatory protein (*hydG*) in DA61770; an asparagine synthetase (*smd_0512*) in DA61771; and a mercury transmembrane transporter (*smd_1285*) in DA61776 (Table 3).

PR-39 exposure led to the acquisition of 15 genetic changes in total, with at least two mutations per clone except for one isolate showing only one mutation. All PR-39-isolated clones, excluding DA61994 and DA61995, acquired mutations in different positions of the stringent starvation protein B-coding gene, *sspB*, leading to amino acid changes, frameshifts, and a change of a stop codon to a sense codon. Another mutation that was present in seven out of eight clones was an SNP in the intergenic region between the cytochrome *c*-coding gene (*smd_1828*) and *smmG*, which encodes a Co/Zn/Cd efflux system membrane fusion protein. This mutation is located in the predicted −10 box of the *smmG* promoter. In addition to these shared changes, mutations were also detected in the *ppa* gene in DA61994, encoding an inorganic phosphatase, and in *sdhA* in DA61995, whose product is a succinate dehydrogenase flavoprotein subunit (Table 3).

Colistin exposure resulted in the selection of 18 mutations among the isolated colonies, where two clones acquired one mutation, three clones had two mutations, two clones had three mutations, and one of them acquired four mutations. The lipopolysaccharide (LPS) transporter protein-coding gene (*lptB*) was mutated in three of the clones, with the same amino acid change (Thr179Pro) in two of them (DA61947 and DA61948) and a change of a stop codon to a sense codon in DA61860. Clones DA62004 and DA61989 had a stop to sense codon shift in the superoxide dismutase-coding gene, *sodB*, whereas the other colistin-isolated clones had amino acid changes in proteins with diverse functions, such as the cell division protein FstW (DA61860), the response regulator RpfG (DA62004), the sensor protein PhoQ (DA61949), or the cyclic AMP receptor protein Crp (DA61950). In DA61948 and DA62004, the same mutation can be found in the predicted promoter region of the *smd_2762* gene, which was also selected under LL-37 selective pressure, suggesting a contribution of this hypothetical protein in the reduced susceptibility to both peptides. Two mutations were also found in DA61860 in the intergenic sequence between *suhB*, encoding an inositol-1-monophosphatase, and *htpX*, encoding an HtpX protease-homologue (Table 3).

### Cross-resistance of AMP-evolved populations to antibiotics is common.

**FIG 1:**
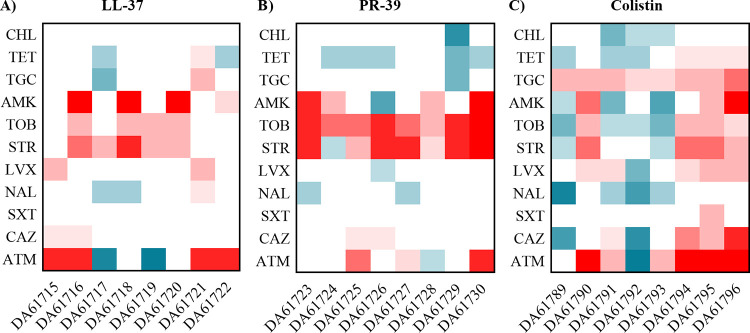
Heat maps representing fold changes in MIC of antibiotics for AMP-resistant populations. Susceptibility to several antibiotics was measured in the S. maltophilia populations evolved in the presence of LL-37 (A), PR-39 (B), and colistin (C). Fold changes were determined using the MIC values of the parental strain D457 as a reference. CHL, chloramphenicol; TET, tetracycline; TGC, tigecycline; AMK, amikacin; TOB, tobramycin; STR, streptomycin; LVX, levofloxacin; NAL, nalidixic acid; STX, trimethoprim-sulfamethoxazole; CAZ, ceftazidime; ATM, aztreonam.

The effect of acquired AMP resistance on the susceptibility of S. maltophilia to antibiotics was assessed by performing MIC assays for several classes of antibiotics for all the evolved populations. Figure 1 shows the changes in MICs of 11 antibiotics. In general, a decreased susceptibility against aminoglycosides was observed for many of the populations that have evolved under LL-37 and PR-39 selection. Some of them also showed an increased resistance to aztreonam. However, two of the LL-37 populations show a 4-fold decrease in the MIC against this beta-lactam, indicating that there is not a direct correlation between resistance to AMPs and aztreonam. For the colistin-evolved populations, there was a high variability among the susceptibility profiles, but interestingly, all of them showed an increase in the MIC of tigecycline, an antibiotic that has been proposed as the drug of choice for treating S. maltophilia infections when trimethoprim-sulfamethoxazole cannot be used (22).

**FIG 2:**
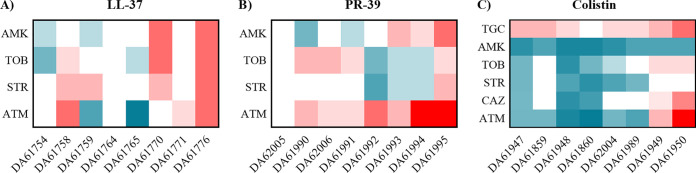
Heat maps representing fold changes in MIC of antibiotics for AMP-resistant isolated clones. Susceptibility to the aminoglycosides amikacin (AMK), tobramycin (TOB), and streptomycin (STR) and the beta-lactam aztreonam (ATM) was measured in the S. maltophilia resistant clones isolated from the populations evolved in the presence of LL-37 (A) and PR-39 (B). Susceptibility to tigecycline (TGC) and ceftazidime (CAZ) were also determined in the colistin-isolated colonies (C). Fold changes were determined using the MIC values of the parental strain D457 as a reference.

The MIC changes of some of the antibiotics that showed variation in the population susceptibility were also determined for the isolated colonies (Fig. 2). Three of the clones from the LL-37-evolved populations showed cross-resistance to aminoglycosides and some of them to aztreonam. Clones DA61759 and DA61765 showed hypersusceptibility to aztreonam similar to that observed for their respective parental populations. Since these clones had only one mutation in the *mraW* gene, this change is responsible for the phenotype. Although DA61758 also harbored a mutation in *mraW*, it also contained a deletion in the TonB-coding gene, which has been reported to be involved in beta-lactam resistance in S. maltophilia (23). The increase in the MICs of aminoglycosides for the PR-39-evolved populations was only observed in some of the isolates. In fact, DA61992, DA61993, and DA61994 show an increased susceptibility to tobramycin and streptomycin. Conversely, most of the PR-39-derived colonies were resistant to aztreonam, even though this phenotype was only observed in three of the parental populations. All of the colistin-resistant clones (except DA61860) maintained an increased MIC of tigecycline, but for the other tested antibiotics a hypersusceptible phenotype was observed in most of the clones. Only the DA61949 and DA61950 clones showed a decreased susceptibility to tobramycin, ceftazidime, and aztreonam. Overall, these results show that AMP resistance in S. maltophilia is associated with cross-resistance to several key classes of antibiotics.

### AMP-resistant mutants generally maintain fitness.

**FIG 3:**
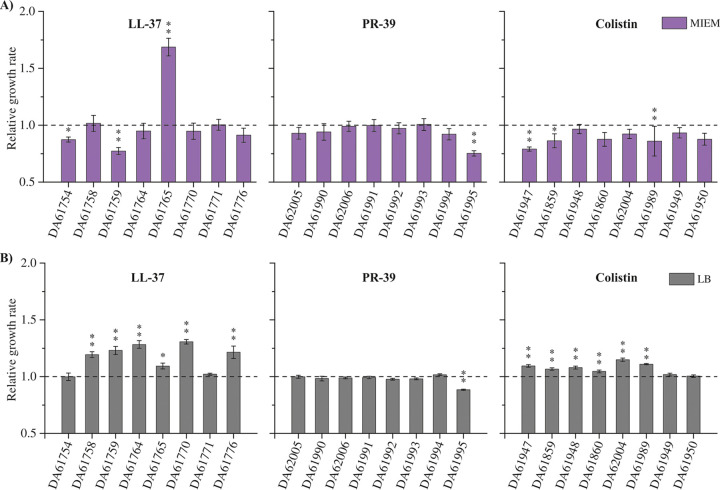
Fitness determination in MIEM and LB growth media. Exponential growth rates were determined by OD_600_ measurements over time for clones isolated from LL-37, PR-39, and colistin-evolved populations in MIEM (A) or LB (B) media. Relative growth rates were calculated using the parental strain D457 as a reference (dotted line). Error bars represent standard deviation for five independent replicates. Statistical significance relative to D457 was assessed by one-way analysis of variance (ANOVA) test (**, *P > *0.0001; *, *P > *0.001).

As the fitness effects of resistance mutations are a key parameter in determining the evolutionary success of resistant bacteria, we examined the fitness of the AMP-resistant mutants by measuring exponential growth rates in MIEM and LB medium (Fig. 3). A growth enhancement was observed for many of the LL-37 and colistin-derived colonies in rich medium (LB). Conversely, we did not observe general significant changes in growth rates in the MIEM conditions. Thus, a few clones displayed a slight growth impairment, except for clone DA61765, which showed an increase of more than 50% in its growth rate. For the PR-39 clones, no fitness costs were detected except for a substantial growth defect in clone DA61995 in both media. These data indicate that AMP resistance is not generally associated with a fitness cost in S. maltophilia. Rather, some fitness improvement was observed in some of the resistant clones.

## DISCUSSION

This study aimed to (i) assess the potential of S. maltophilia to acquire AMP resistance, (ii) identify the resistance mutations, (iii) examine cross-resistance to other antimicrobials, and (iv) determine if resistance confers a fitness cost. S. maltophilia adapted both phenotypically and genotypically to each AMP, and all populations showed a reduced susceptibility to the peptide used in the evolution, and often cross-resistance to other peptides and certain classes of conventional antibiotics. Most importantly, AMP resistance could develop very rapidly to all three tested AMPs, and after only 165 generations of growth in the presence of AMP, resistance levels were increased 2- to >8-fold, depending on the mutant and AMP. Interestingly, even though LL-37 and PR-39 are both mammalian cathelicidins, they showed marked differences in evolutionary outcome. It was previously described that diverse cathelicidins from several species have different antimicrobial and immunomodulator activities (24), and our data support the notion that different compounds within the same family of AMPs can have different functions and thereby influence resistance selection (see below). Furthermore, there was extensive cross-resistance between the AMPs. Thus, all LL-37-selected populations displayed cross-resistance to colistin, all PR-39-selected populations showed cross-resistance to both LL-37 and colistin, and some colistin-resistant populations had a reduced susceptibility to LL-37 and PR-39. Thus, cross-resistance between the different classes of AMPs is common, as has also been shown for human defensin AMPs that are part of the host-innate immune system (25–27).

**FIG 4:**
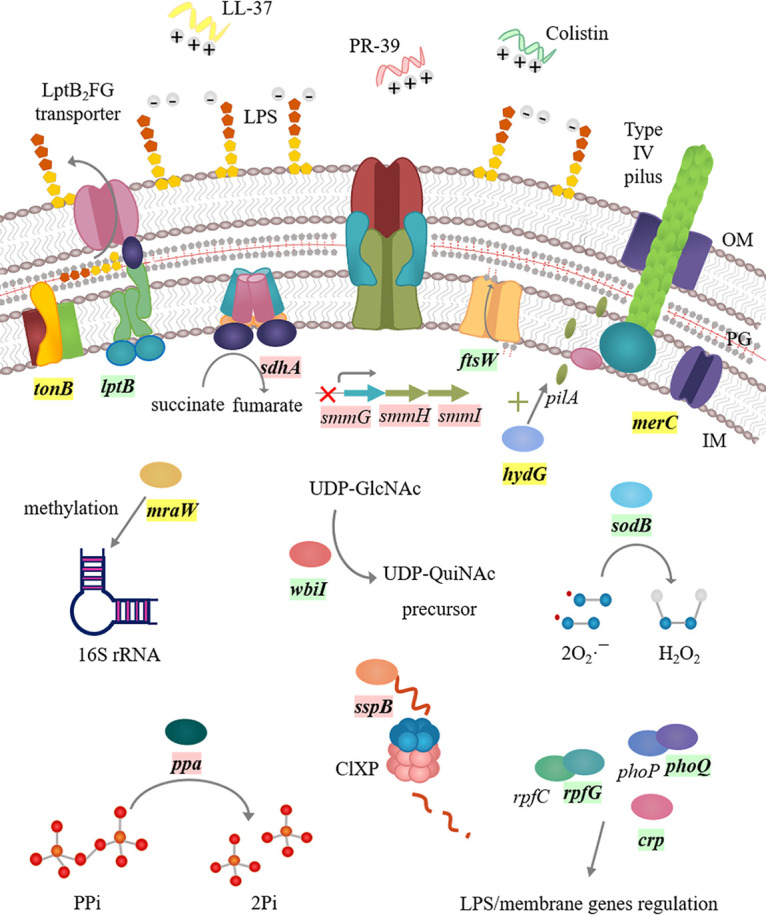
Schematic drawing of S. maltophilia acquired AMP resistance mechanisms. LL-37, PR-39 and colistin (all positively charged) interact with the negatively charged bacterial membrane to exert their action. Mutated genes are marked in bold, being specifically selected after LL-37 (yellow), PR-39 (pink), or colistin (light green) exposure. S. maltophilia can modulate the membrane charge and permeability, changing its composition and reducing the membrane negative charge (*lptB*, *ftsW*, and *wbiI*). The activity of proteases such as ClpXP contributes to the regulation of stress-related genes, as well as that of proteases, such as metalloproteases, that degrade AMPs. S. maltophilia can also sense and respond to AMP presence through several regulators and enzymes involved in the expression of genes that can modify the bacterial membrane and lead to resistance (*phoQ*, *rpfG*, *crp*, *mraW*, and *hydG*). The bacterium can act against the AMP-mediated disruption of the electron transport chain (*sdhA*) or the ion flows across the membrane as inorganic phosphate (Pi) (*ppa*). The production by AMPs of hydroxyl radicals such as colistin can be counteracted by enzyme-coding genes such as *sodB*. The expression of multidrug efflux pumps can lead to AMP extrusion outside the cell (*smmGHI*), decreasing the accumulation of the drug. IM, inner membrane; OM, outer membrane; PG, peptidoglycan; LPS, lipopolysaccharide.

LL-37 acts as a pore-forming toxin that interacts in the bacterial inner membrane (5); colistin interacts with the outer membrane, displacing Ca^2+^ and Mg^2+^ from the phosphate groups of membrane lipids, leading to disruption of the outer cell membrane and death (9); and PR-39 is thought to act in the cytoplasm, inhibiting DNA and protein synthesis (28). Since the three AMPs examined act on different targets, one would expect to find different resistance mechanisms for each peptide. This idea was confirmed by the WGS data, in which a total of 48 mutations were identified after the four evolution experiments (LL-37, PR-39, colistin, and control), with little overlap in the mutational spectra between the AMPs. Nevertheless, and although the selected mutations were different, the cross-resistance exhibited by several of the mutants indicates that, despite presenting different targets, acquiring resistance to one AMP may compromise the activity of all of them. The potential roles of some of them in AMP resistance are summarized in Table 3, Fig. 4, and below.

LL-37- and colistin-resistant clones showed a high heterogeneity among the mutated genetic determinants, and many of them are expected to result in modifications of the S. maltophilia envelope. For instance, some colistin mutants acquired mutations in the *lptB*, *ftsW*, and *wbiI* genes that can result in modification of the membrane LPS content and changes in the peptidoglycan composition, rendering the bacterium less susceptible to colistin and other AMPs (29–32). Modifications in the bacterial membrane that reduce or inhibit the AMP-membrane interaction can be achieved indirectly through altered regulators and/or two-component systems. Among them, the *hydG* gene encoding the regulatory protein PilR, which regulates the expression of the type IV pilus assembly protein PilA, was mutated in one LL-37 isolate (33). The absence of PilA has been associated with a reduced membrane stability (34). Thus, the acquired mutation could alter the expression of *pilA* and affect membrane permeability as a protection mechanism toward LL-37 killing. Mutations in the genes encoding the regulators PhoQ, RpfG, and Crp, which control the expression of several proteins involved in LPS modification, pathogenicity, and biosynthetic enzymes for extracellular polysaccharides (35–41), were found in some colistin-resistant clones. Besides altering the expression of genes that encode membrane/LPS-associated functions, they could also affect the expression of genes that alleviate the cellular damage caused by colistin. Three LL-37-resistant clones had mutations in the methyltransferase H-coding gene, *mraW*, which plays a leading role in the fine-tuning of the function of the ribosomal P-site and start codon selection (42, 43). The specific methylation of 16S rRNA carried out by MraW can lead to the modification of many bacterial physiological processes, including antimicrobial susceptibility.

Besides acting in the bacterial membrane, colistin induces killing by hydroxyl radical production in Gram-negative bacteria (44). A mutation in the superoxide dismutase-coding gene *sodB* was selected in two of the colistin resistant isolates as a potential defense mechanism against the induction of hydroxyl radical production by colistin.

Mutations in genes encoding transport functions (*smd_0947*, *smd_1285*, and *smd_0008*) were also found in LL-37-selected clones. While no information about SMD_0947 is available, SMD_1285 is a homologue of MerC, which is associated with mercury resistance (45). Mutations in *merC* have been found in S. maltophilia clinical isolates from a patient with CF that had undergone several antibiotic treatments (46). The *smd_0008* gene encodes the TonB protein, which is part of the bacterial iron uptake system, (47). Since many antimicrobial agents cross the bacterial membrane through TonB-dependent receptors, the mutation in this protein could lead to a reduced interaction or uptake of LL-37 (48, 49).

A majority of the PR-39-resistant clones shared the same mutations. Thus, six clones had genetic changes in the *sspB* gene. The stringent starvation protein, SspB, activates the ATPase activity of ClpX, a component of the ClpXP protease, hence enhancing its proteolytic activity. Together with SsrA (50), which introduces a degradation tag to proteins that are stalled on the ribosome, SspB regulates several proteins. Besides, SspB also delivers substrates that are not SsrA-tagged (51). Hence, the *sspB* mutations probably influence ClpX-mediated degradation of several proteins and alter the expression of genes related to cellular stress. In agreement with our findings, ClpXP contributes to antibiotic tolerance and peptide resistance in different bacterial species (52, 53). Extrusion of AMPs by an energy-dependent efflux system constitutes an important AMP resistance mechanism (54–56), and exposure to antibiotics and biocides can select for mutation-driven overexpression of efflux pumps in S. maltophilia (15, 16, 57). Here, a mutation located in the predicted −10 box of the *smmG* promoter, which encodes a Co/Zn/Cd efflux system membrane fusion protein, was found in seven of the PR-39 resistant clones. This membrane fusion protein belongs to the resistance nodulation division (RND) family of MDR efflux pumps (58), and increased expression of this efflux system could cause extrusion of PR-39 from the cell. Mutations in *ppa* and in *sdhA* were also found in two PR-39-resistant clones. Ppa is an inorganic pyrophosphatase that catalyzes the hydrolysis of pyrophosphate to two phosphate ions. Interaction of AMPs with the bacterial membrane often results in a loss of control over ions flow, including that of inorganic phosphate ions, across the membrane (10), and the *ppa* mutation could act to restore the inorganic phosphate ion flow. Finally, the succinate dehydrogenase SdhA participates in both the TCA cycle and the electron transport chain (ETC) (59). AMPs are able to disrupt the appropriate flow of electrons through the ETC, releasing oxidative species into the bacterial periplasm and permeabilizing the cytoplasmic membrane (60–62). The mechanism by which changes in SdhA confers PR-39 resistance is unclear, but it might be involved in the proper ETC functioning.

One potential problem with using AMPs clinically is the possibility that resistance to these compounds might be associated with cross-resistance to other classes of antimicrobials (25, 63–65). We hypothesize that the genetic changes altering the membrane composition or membrane permeability, such as in *lptB*, *ftsW*, or *hydG*, generates low susceptibility not only to AMPs, but also to antibiotics. The majority of the LL-37- and PR-39-evolved populations displayed cross-resistance to aminoglycosides, and some of them to the beta-lactam aztreonam. The alteration of the bacterial outer membrane as a response to AMP exposure can be responsible for this cross-resistance, since aminoglycosides are thought to enter the cell through a self-promoted uptake mechanism, interacting with and disrupting the outer membrane (66). This could also affect the uptake and action of beta-lactam antibiotics, such as aztreonam. Colistin-evolved populations displayed different phenotypes to the tested antibiotics, where approximately half of them were resistant, and the other half showed a hypersusceptible profile. Remarkably, all the evolved populations displayed cross-resistance against tigecycline. For the isolated colonies, we could not observe the same susceptibility patterns for all the clones as those of the populations, and only some isolates displayed cross-resistance to the tested antibiotics. This indicates that the evolved populations were heterogenous and that no single clone had swept the population. Six of the colistin-resistant clones showed collateral susceptibility to aminoglycosides (all of them to amikacin) and beta-lactams. This collateral sensitivity could be exploited in clinics through combination therapy or cycling of different antimicrobial compounds (67).

It is widely accepted that antibiotic resistance typically confers a reduced fitness in the absence of drug (68) but data regarding AMP resistance are scarce and very few studies have addressed this question, obtaining different results (25, 69–71). While Spohn et al. observed a general fitness cost for most of the AMP-adapted lines (71), the study of Kubicek-Sutherland et al. reported a fitness gain in MIEM medium but a growth impairment in other media (25). We estimated the fitness of all the isolated colonies by measuring exponential growth rates in drug-free MIEM and LB media. Notably, and opposite to what could be expected, we observed a significant fitness increment for some of the LL-37- and colistin-isolated colonies in rich LB medium, while a moderate fitness cost was observed for some of them in MIEM medium, except for one LL-37 isolate (DA61765) that showed a considerable fitness increment and a unique mutation in *mraW* (in a different location to those present in DA61758 and DA61759). Strain DA61765 had a deletion mutation at the end of MraW, whereas strains DA61758 and DA61759 had deletions in the middle of the protein, which could potentially explain their different phenotypic effects. Methylation by MraW results in changes in the cellular growth properties in Escherichia coli (42), and it is possible that this alteration is the reason for its fitness improvement. DA61995 is the only PR-39-resistant isolate which showed a pronounced fitness reduction in both media. Succinate dehydrogenase genes have been reported to be involved in fitness compensation of the metabolic cost of antibiotic resistance in E. coli (72). Thus, the mutation in *sdhA* in strain DA61995 might affect the fitness of this mutant. Together, these data indicate that acquisition of AMP resistance, is not only cost free but can even improve S. maltophilia fitness, a characteristic that may favor the dissemination and persistence of these resistant mutants in the absence of selective pressure. A key question regards the relevance of *in vitro* selection and if AMP resistance can also be acquired *in vivo*. It is notable that the levels of LL-37 and PR-39 found in humans and pigs during an infection are close to the concentrations used at the beginning of the evolution experiment, implying that resistance selection in S. maltophilia could occur *in vivo* and generate mutants that can resist key components of our innate immunity (73–75). These findings, combined with the ease by which high-level AMP resistance can be acquired and the high prevalence of cross-resistance to several clinically important antibiotic classes, warrant a strict surveillance of resistance evolution to AMPs in clinical use.

## MATERIALS AND METHODS

### Bacterial strains and growth conditions.

Bacterial strains in this study originate from the S. maltophilia clinical isolate D457 (76). All the experiments were performed at 37°C. Liquid MIEM medium without NaCl, as described in Dorschner et al. (77), was used for the experimental evolution and fitness determination experiments. Lysogeny broth (LB) was used for fitness assays, and Mueller-Hinton II (cation-adjusted) liquid and agar was used for antibiotic susceptibility determination. LL-37 (LLGDFFRKSKEKIGKEFKRIVQRIKDFLRNLVPRTES) and PR-39 (RRRPRPPYLPRPRPPPFFPPRLPPRIPPGFPPRFPPRFP-NH2) were synthesized by Innovagen AB, and colistin was obtained from Sigma-Aldrich.

### AMP experimental evolution by serial passage.

The experimental evolution assay was initiated with the S. maltophilia D457 strain growing in the presence of a peptide concentration that caused a 30% reduction in the bacterial growth. These concentrations were determined by performing growth curves in MIEM medium and measuring the optical density at 600 nm (OD_600_) using a Bioscreen C plate reader (Oy Growth Curves AB, Ltd.). The starting concentrations were 50 mg/liter for LL-37, 2 mg/liter for PR-39, and 0.25 mg/liter for colistin. A control assay without any compound was also performed. An S. maltophilia D457 overnight culture was used for starting the evolution experiment, with eight independent replicates for each experimental condition. The assay was started by inoculating 1 μl of bacterial culture in 100 μl of MIEM, with or without the AMP, in round-bottomed 96-well plates (Nunc; Thermo Fisher Scientific) that were incubated at 37°C with shaking. Serial passages were performed every 24 h by transferring 1 μl of cell culture in 100 μl of fresh MIEM. Every 3 days, the peptide concentration was increased by 50% if growth allowed. If bacterial cultures showed poor/no growth, the peptide concentration used at that evolution step was maintained, or even reduced by 50%, for another cycle in order to continue the passage. The 96-well plates were saved at −80°C by adding dimethyl sulfoxide (DMSO) to a final concentration of 10% in each well, allowing the recovery and reinoculation in cases where bacterial growth was poor or absent. This procedure was performed during 25 days, during which the LL-37 concentration reached 168.75 mg/liter for two replicates and 112.5 mg/liter for six replicates (3.3- and 2.25-fold higher than the starting concentration), the PR-39 concentration reached 51.3 mg/liter for three replicates and 34.2 mg/liter for five replicates (25.25- and 17.1-fold higher than the starting concentration), and the colistin concentration reached 1.59 mg/liter for six replicates and 1.06 mg/liter for two replicates (6.36- and 4.24-fold higher than the starting concentration) (Fig. S1). At the end of the experiment, single clones were isolated from the population of each independent evolution experiment (eight colonies per peptide and four colonies from the control experiment) for further analysis.

### DNA extraction, WGS, and identification of mutations.

Genomic DNA from the isolated colonies was extracted at the end of the assay using the Qiagen Genomic-tip 100/G together with the genomic DNA buffer kit (Qiagen) following the manufacturer’s protocol. The quality of the extracted DNA was assessed by electrophoresis in agarose gel, and DNA quantity was measured with a Qubit 2.0 fluorometer. WGS was performed with a MiSeq instrument (Illumina) in-house at the department of Medical Biochemistry and Microbiology. The libraries were prepared with the Nextera XT DNA library preparation kit, and the sequencing was done with a V3 600-cycle reagent cartridge. The sequencing was achieved to an average of at least 30× coverage. Data analysis was accomplished with CLC Genomics Workbench software (Qiagen), and the genetic changes were identified through the mapping of the obtained reads to the S. maltophilia D457 reference genome (GenBank accession number NC_017671.1). The given variants were then filtered against those of the D457 laboratory wild-type strain.

### Antimicrobial susceptibility assays.

The MICs of the antimicrobial peptides were determined by the double-dilution method in round-bottomed 96-well plates (Nunc; Thermo Fisher Scientific) in liquid MIEM medium at 37°C. MICs of antibiotics were determined using MIC test strips (Liofilchem and AB bioMérieux) on Mueller-Hinton II agar plates at 37°C.

### Fitness cost measurement.

Each independent colony isolated from the serial-passage experiments, as well as the parental strain D457, were used for this assay. Fitness cost determination was performed as described in Kubicek-Sutherland et al. (25). Briefly, samples were grown for 16 h at 37°C in a Bioscreen C plate reader (Oy Growth Curves AB, Ltd.), taking OD_600_ measurements of five technical replicates every 4 min. Maximum growth rates were calculated using the OD_600_ values in the exponential growth phase using the Bioscreen Analysis Tool (BAT) 2.0 software (78). Relative growth rates were obtained by dividing the values of each independent colony by those derived from the D457 wild-type strain under the same conditions.
