# Supplementary figures and images for: Antimicrobial Peptide Exposure Selects for Resistant and Fit Stenotrophomonas maltophilia Mutants That Show Cross-Resistance to Antibiotics
Source: mSphere. 2020 Sep 30;5(5):e00717-20. doi: 10.1128/mSphere.00717-20 (PMC7529437; doi:10.1128/mSphere.00717-20)

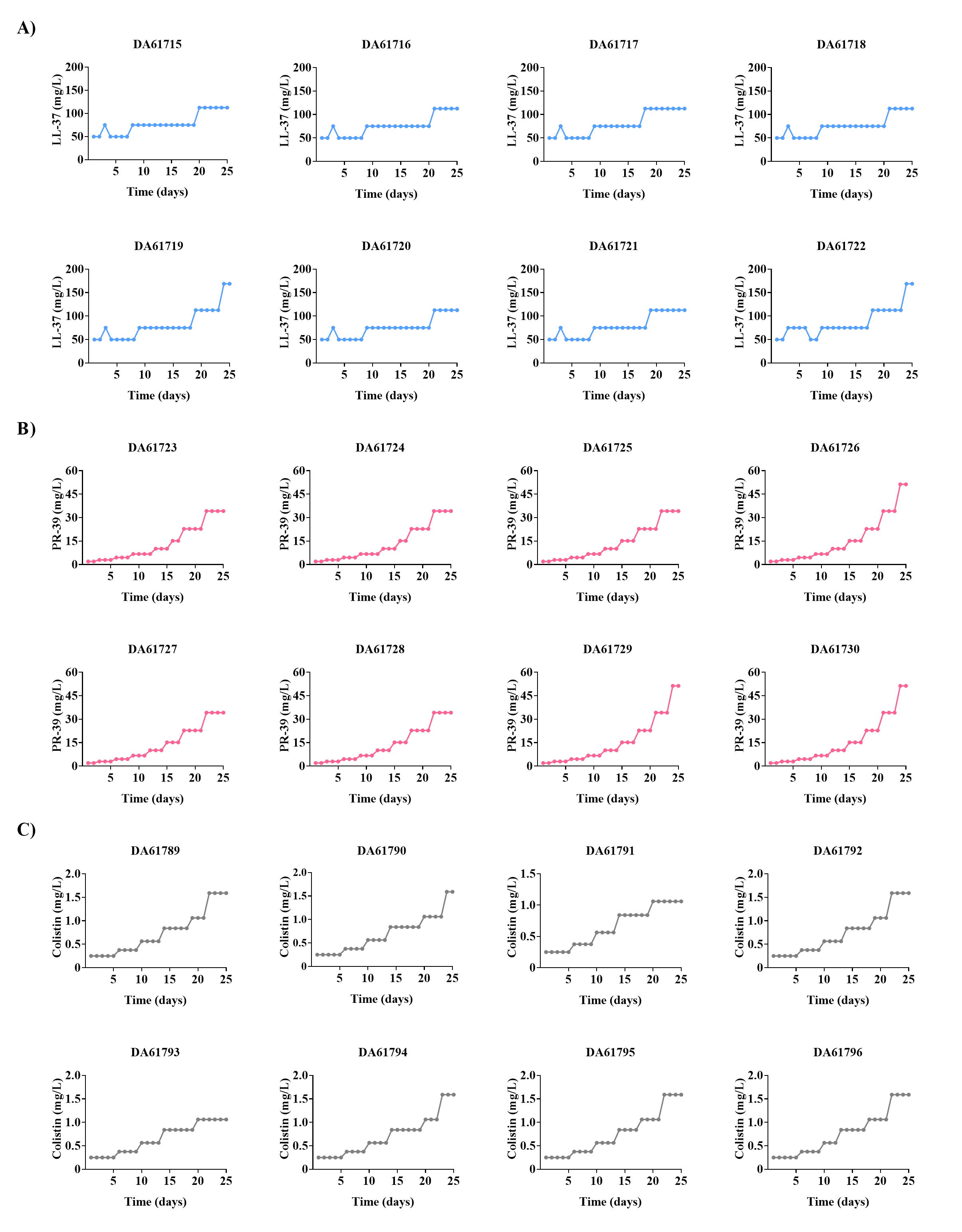

Supplement: FIG S1 [file mSphere.00717-20-sf001.tif]
